# Elucidation of the Mechanisms and Molecular Targets of Yiqi Shexue Formula for Treatment of Primary Immune Thrombocytopenia Based on Network Pharmacology

**DOI:** 10.3389/fphar.2019.01136

**Published:** 2019-10-01

**Authors:** Yunyao Jiang, Nan Liu, Shirong Zhu, Xiaomei Hu, Dennis Chang, Jianxun Liu

**Affiliations:** ^1^Xiyuan Hospital, China Academy of Chinese Medical Sciences, Beijing, China; ^2^School of Pharmaceutical Sciences, Institute for Chinese Materia Medica, Tsinghua University, Beijing, China; ^3^Beijing Key Laboratory of TCM Pharmacology, Xiyuan Hospital, China Academy of Chinese Medical Sciences, Beijing, China; ^4^Department of PK- PD, Beijing Increase Research for Drug Efficacy and Safety Co., Ltd, Beijing, China; ^5^NICM Health Research Institute, Western Sydney University, Westmead, NSW, Australia

**Keywords:** Yiqi Shexue formula, primary immune thrombocytopenia, network pharmacology, mechanism, target gene, pathway

## Abstract

Yiqi Shexue formula (YQSX) is traditionally used to treat primary immune thrombocytopenia (ITP) in clinical practice of traditional Chinese medicine. However, its mechanisms of action and molecular targets for treatment of ITP are not clear. The active compounds of YQSX were collected and their targets were identified. ITP-related targets were obtained by analyzing the differential expressed genes between ITP patients and healthy individuals. Protein–protein interaction (PPI) data were then obtained and PPI networks of YQSX putative targets and ITP-related targets were visualized and merged to identify the candidate targets for YQSX against ITP. Gene ontology and Kyoto Encyclopedia of Genes and Genomes pathway analysis were carried out. The gene-pathway network was constructed to screen the key target genes. In total, 177 active compounds and 251 targets of YQSX were identified. Two hundred and thirty differential expressed genes with an *P* value < 0.005 and |log2(fold change)| > 1 were identified between ITP patient and control groups. One hundred and eighty-three target genes associated with ITP were finally identified. The functional annotations of target genes were found to be related to transcription, cytosol, protein binding, and so on. Twenty-four pathways including cell cycle, estrogen signaling pathway, and MAPK signaling pathway were significantly enriched. MDM2 was the core gene and other several genes including TP53, MAPK1, CDKN1A, MYC, and DDX5 were the key gens in the gene-pathway network of YQSX for treatment of ITP. The results indicated that YQSX’s effects against ITP may relate to regulation of immunological function through the specific biological processes and the related pathways. This study demonstrates the application of network pharmacology in evaluating mechanisms of action and molecular targets of complex herbal formulations.

## Introduction

Primary immune thrombocytopenia (ITP) is the most common autoimmune cytopenia characterized by transient or persistent decreased platelet count (Comont et al., 2017; Castro, 2017). The occurrence of ITP results from the generation of anti-platelet autoantibodies against platelet membrane glycoproteins finally leading to the destruction of platelets in the reticuloendothelial system, especially in the spleen (Zhang et al., 2015). ITP patients have an increased risk of bruising, cutaneous bleeding, and infrequently serious bleeding including intracranial hemorrhage (Lin et al., 2017). In addition, the quality of life of ITP patients is affected as a result of the physical and psychological symptoms, discomfort, fear, reduced social activity, and reduced ability to work (López et al., 2015). The standard therapy for newly diagnosed ITP patients is to stop bleeding and increase platelet counts using pharmaceutical medicines (Kühne, 2015). However, these treatments are often accompanied by harmful side effects, which tend to be more evident with the time of treatment. In addition, the high treatment costs cast a heavy financial burden to ITP patients, especially those with severe forms of the disease (Khellaf et al., 2011).

In recent years, traditional Chinese medicine (TCM) has been regarded as a potential effective auxiliary strategy to treat the chronic diseases, including ITP (He et al., 2015). Huang et al. (2013) reported that a modified Chinese herbal formula, Zi-Ying-Jiang-Huo-Tang (Phellodendri Combination) cured a 4-year-old ITP patient who did not respond to a 7-month first-line conventional treatment of steroids and intravenous immunoglobulin, and no recurrence of the disease or side effects of the treatment were found during the 12-month follow-up period. Yang et al. (2017) reported that imbalance of Th1/Th2 and Th17/Treg cells play a crucial role in the pathogenesis of chronic ITP and that Yiqi Tongyang Decoction significantly regulated the dynamics of Th1/Th2 and Th17/Treg equilibria.

Yiqi Shexue formula (YQSX), an improved formula of Bazhen decoction (BZD), is a mixture of 9 Chinese medicine extracts including *Ginseng Radix et Rhizoma* (GRR, the dried root and rhizome of *Panax ginseng* C. A. Mey.), *Poria* [P, the dried sclerotium of *Poria cocos* (Schw.) Wolf], *Atractylodis Macrocephalae Rhizoma* (AMR, the dried rhizome of *Atractylodes macrocephala* Koidz.), *Glycyrrhizae Radix et Rhizoma* (RRG, the dried root and rhizome of *Glycyrrhiza uralensis* Fisch), *Angelicae Sinensis Radix* [ASR, the dried root of *Angelica sinensis* (Oliv.) Diels], *Chuanxiong Rhizoma* (CR, the dried rhizome of *Ligusticum chuanxiong* Hort.), *Paeoniae Radix Alba* (PRA, the dried root of *Paeonia lactiflora* Pall.), *Rehmanniae Radix Praeparata* (RRP, the dried root of *Rehmannia glutinosa* Libosch.), and *Asini Corii Colla* (ACC, the product of hide of *Equus asinus* L.). In TCM, BZD is frequently used to treat the deficiency of *qi* and *blood* which is characterized by many symptoms, including anemia, asthenia, dizziness, chronic abscess, fatigue, irregular menstruation, palpitations, fatigue of the muscles, and pale complexion (Song et al., 2014). It has been reported that BZD could substantially promote the proliferation of bone marrow hematopoietic cells in anemic mice (Tian et al., 2016). YQSX is formulated based on BZD with one additional medicine (ACC). ACC is obtained from *Equus asinus Linnaeus* and has been widely used to promote health in China for life cultivation and clinical hematic antanemic therapy as a health food and TCM for more than 2,000 years. And early evidence has shown that ACC possesses a therapeutic effect in treating various hematologic diseases, such as anemia, aleucocytosis, and thrombopenia (Wang et al., 2014). In TCM, YQSX has been suggested to be able to invigorate *spleen*, replenish *qi*, nourish *blood*, and promote blood circulation and is traditionally used to treat ITP in clinical practice. However, the mechanisms of action and molecular targets of YQSX for treatment of ITP are not clear, which is the main factor limiting its wider use.

In TCM, complex herbal formulations that consist of multiple herbs are used and these complex chemical mixtures include numerous potential bioactive components that can interact with multiple therapeutic targets. This multi-component, multi-target, and multi-pathway approach may be ideal for the treatment of diseases with complex pathophysiology and therapeutic targets, but also present a tremendous challenge in understanding of the interactions between various components, their mechanisms of action and molecular targets. Liu et al. (2016) proposed the concept of Network Pharmacology in an attempt to solve these problems. Network pharmacology is a novel approach that combines system network analysis and pharmacology. It could be used to elucidate the synergistic effects among compounds and potential mechanisms of multi-component and multiple target drugs at the molecular level through the networks of compound–compound, compound–target, and target–disease. Network pharmacology would facilitate the understanding of the interactions among the compounds, genes, proteins, and diseases and is suitable for the study of complex TCM formulations (Xu et al., 2017; Zeng et al., 2017). Chen et al. (2018) explored potential mechanism of Jiawei Foshou San on endometriosis using a network pharmacology approach. The underlying action mechanism of Wu-Tou decoction in rheumatoid arthritis was expounded by network pharmacology prediction and experimental verification (Guo et al., 2017). The potential mechanism between Danggui-shaoyao-san and neurodegenerative disorders was deciphered through a network pharmacology approach (Luo et al., 2016). The research group of Shao Li elucidated anti-rheumatic mechanisms of Qing-Luo-Yin and investigated its possible interactions with methotrexate using an integrating strategy coupled with network pharmacology and metabolomics techniques (Zou et al., 2018).

In the present study, a network pharmacology approach was used to explore the mechanisms of action and molecular targets of YQSX for treatment of ITP. The active compounds of YQSX and their targets were firstly identified using drugbank database. Then ITP-related targets were obtained by analyzing the differential expressed genes between ITP patients and healthy individuals. The mechanisms of action underlying YQSX for the treatment of ITP were analyzed by gene ontology (GO) and pathway analysis.

## Materials and Methods

### Active Ingredients Screening

We identified the chemical composition of YQSX from Traditional Chinese Medicine Systems Pharmacology Database and Analysis Platform (Ru et al., 2014) (TCMSP, http://lsp.nwu.edu.cn/tcmsp.php) and select candidate compounds which has oral bioavailability (OB) ≥ 30% and drug-likeness (DL) ≥ 0.18 (Li et al., 2015). One hundred and sixty eligible compounds were obtained, 22 in GRR, 15 in P, 7 in AMR, 92 in RRG, 2 in ASR, 7 in CR, 13 in PRA, and 2 in RRP. However, 8 compounds including ferulic acid, ligustilide, senkyunolide C, and leonuride which were not found in the database have been selected as they have the pharmacological activity on ITP treatment. Additionally, 19 amino acids including aspartic acid, threonine, and serine in ACC have been reported to process relevant pharmacological properties and were also included (Wang et al., 2014). Eventually, 177 candidate compounds were obtained in total after the duplications were removed.

### Identification of Potential Targets

The 177 candidate compounds were imported into the DrugBank database (Law et al., 2014) (https://www.drugbank.ca/) to identify the corresponding targets of YQSX. One hundred and forty-eight compounds were finally selected after removing 29 compounds which did not link to any targets. And the targets of 148 compounds were collected. Two thousand one hundred and seventy-seven targets were identified, 204 in GRR, 28 in P, 20 in AMR, 1272 in RRG, 106 in ASR, 36 in CR, 99 in PRA, 58 in RRP, and 354 in ACC. A total of 251 targets were collected after removing duplication.

### ITP-Related Targets

The differential expressed genes of ITP patients were obtained from GEO database (https://www.ncbi.nlm.nih.gov/geo/, Series: GSE574, Samples: GSM8814, GSM8815, GSM8816, GSM8817). Genes with a *P* value < 0.005 and |log 2(fold change)| > 1 were considered to be of significantly differential expression and ITP-related targets.

### Network Construction

The compound-target network of YQSX was constructed and visualized using Cytoscape 3.5.1 software. PPI data were obtained from Database of Interacting Proteins (DIP™), Biological General Repository for Interaction Datasets (BioGRID), Human Protein Reference Database (HPRD), IntAct Molecular Interaction Database (IntAct), Molecular INTeraction database (MINT), and biomolecular interaction network database (BIND) using the plugin Bisogenet (Martin et al., 2010) of Cytoscape 3.5.1 software. The PPI networks of YQSX putative targets and ITP-related targets were visualized with Cytoscape software.

### Network Merge

The PPI networks of YQSX putative targets and ITP-related targets were merged with Cytoscape software. And the nodes with topological importance in the interaction network were screened by calculating Degree Centrality (DC), Betweenness Centrality (BC), Closeness Centrality (CC), Eigenvector Centrality (EC), Local average connectivity-based method (LAC), and Network Centrality (NC) with the Cytoscape plugin CytoNCA. These parameters represent the topological importance and they have been reported about their definitions and computational formulas and used in network pharmacology and systems pharmacology (Tang et al., 2015).

### Bioinformatic Analysis

GO analysis with the biological process, cellular component, and molecular function was carried out using the Database for Annotation, Visualization and Integrated Discovery (DAVID, https://david.ncifcrf.gov, v6.8) (Huang et al., 2009). Functional categories were enriched within genes (FDR < 0.05) and the top 20 GO functional categories were selected. DAVID that assigned Kyoto Encyclopedia of Genes and Genomes (KEGG) database was used for pathway analysis. Pathways that had significant changes of FDR < 0.05 were identified for further analysis. The genes that significantly regulated pathways were selected for gene-pathway network analysis. The gene-pathway network was constructed to screen the key target genes that YQSX treated ITP.

## Results

### Compound-Target Network Analysis

**Figure 1:**
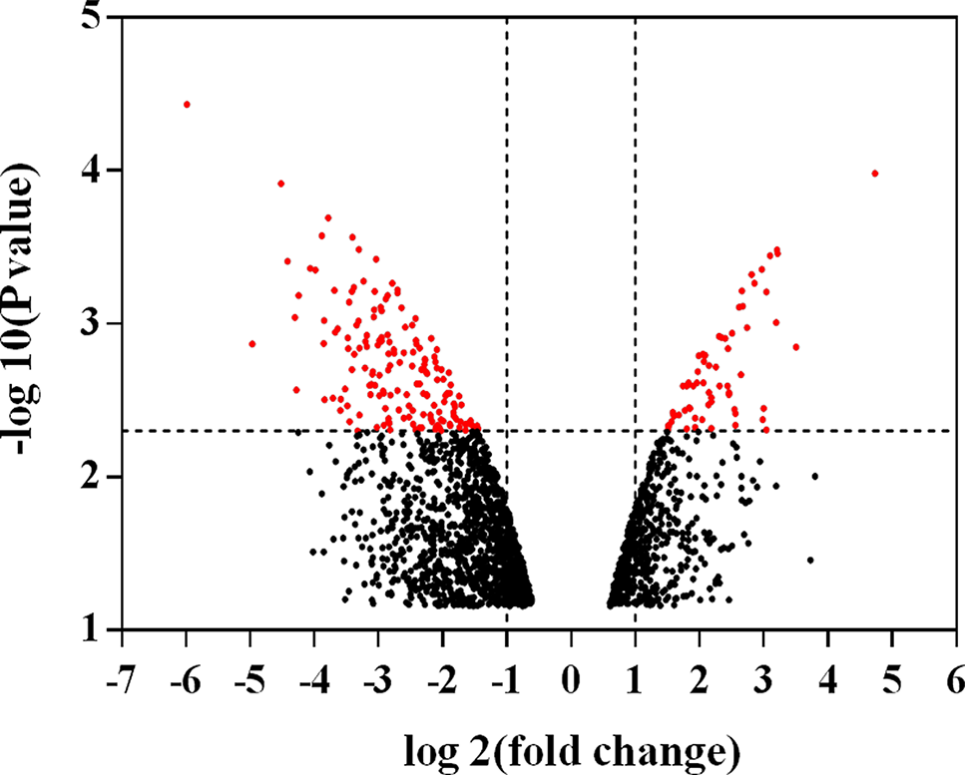
Volcano plot of differentially expressed genes. The abscissa represents the fold changes in gene expression and the ordinate represents the statistical significance of the variations in gene expression. The red dots represent significantly differentially expressed genes.

One hundred and forty-eight compounds of YQSX (**Table 1**) were finally selected as the candidate compounds. And 230 ITP-related targets were identified from GEO database. As shown in **Figure 1**, a volcano plot was created to show the distribution of differentially expressed genes, which were represented by the red dots in the plot.

**Table 1:** The final selected compounds in YQSX for analysis.

ID	Name	OB	DL	Source	ID	Name	OB	DL	Source
MOL000449	Stigmasterol	43.83	0.76	GRR, ASR, RRP	MOL004908	1,3-dihydroxy-8,9-dimethoxy-6-benzofurano[3,2-c]chromenone	62.9	0.53	RRG
MOL000358	ß-sitosterol	36.91	0.75	GRR, ASR, PRA	MOL004910	(2R)-7-hydroxy-2-(4-hydroxyphenyl)chroman-4-one	71.12	0.18	RRG
MOL003648	Inermin	65.83	0.54	GRR	MOL004911	Glabrene	46.27	0.44	RRG
MOL005317	Deoxyharringtonine	39.27	0.81	GRR	MOL004912	Glabrone	52.51	0.5	RRG
MOL005320	Arachidonate	45.57	0.2	GRR	MOL004913	Hedysarimcoumestan B	48.14	0.43	RRG
MOL005321	Frutinone A	65.9	0.34	GRR	MOL004914	Glabridin	53.25	0.47	RRG
MOL005356	Girinimbine	61.22	0.31	GRR	MOL004915	Eurycarpin A	43.28	0.37	RRG
MOL005376	Panaxadiol	33.09	0.79	GRR	MOL004924	(-)-Medicocarpin	40.99	0.95	RRG
MOL005384	Suchilactone	57.52	0.56	GRR	MOL004935	Sigmoidin B	34.88	0.41	RRG
MOL000787	Fumarine	59.26	0.83	GRR	MOL004941	Glabranin	52.9	0.31	RRG
MOL002879	Diop	43.59	0.39	GRR	MOL004945	Isobavachin	36.57	0.32	RRG
MOL000422	Kaempferol	41.88	0.24	GRR, PRA, RRG	MOL004948	Isoglycyrol	44.7	0.84	RRG
MOL005308	Aposiopolamine	66.65	0.22	GRR	MOL004949	Isolicoflavonol	45.17	0.42	RRG
MOL005344	Ginsenoside Rh2	36.32	0.56	GRR	MOL004957	Isoformononetin	38.37	0.21	RRG
MOL005348	Ginsenoside Rh4	31.11	0.78	GRR	MOL001484	Inermine	75.18	0.54	RRG
MOL005318	Dianthramine	40.45	0.2	GRR	MOL004959	1-Methoxyphaseollidin	69.98	0.64	RRG
MOL005399	Daucosterol	36.91	0.75	GRR	MOL004993	8-prenylated eriodictyol	53.79	0.4	RRG
MOL000676	Dibutyl Phthalate	64.54	0.13	GRR, PRA	MOL004961	Quercetin der.	46.45	0.33	RRG
MOL000273	(3ß,16α)-3,16-Dihydroxylanosta-7,9(11),24-trien-21-oic acid	30.93	0.81	P	MOL004966	7,2′,4′-trihydroxy-5-methoxy-3-arylcoumarin	83.71	0.27	RRG
MOL000275	Trametenolic acid	38.71	0.8	P	MOL000497	Licochalcone A	40.79	0.29	RRG
MOL000279	Cerevisterol	37.96	0.77	P	MOL004974	3′-Methoxyglabridin	46.16	0.57	RRG
MOL000282	Stellasterol	43.51	0.72	P	MOL004978	4′-Methoxyglabridin	36.21	0.52	RRG
MOL000283	Ergosterol peroxide	40.36	0.81	P	MOL004980	Inflacoumarin A	39.71	0.33	RRG
MOL000296	Hederagenin	36.91	0.75	P	MOL004985	Icos-5-enoic acid	30.7	0.2	RRG
MOL000022	14-acetyl-12-senecioyl-2E,8Z,10E-atractylentriol	63.37	0.3	AMR	MOL004988	Kanzonol F	32.47	0.89	RRG
MOL000033	(3S,8S,9S,10R,13R,14S,17R)-10,13-dimethyl-17-[(2R,5S)-5-propan-2-yloctan-2-yl]-2,3,4,7,8,9,11,12,14,15,16,17-dodecahydro-1H-cyclopenta[a]phenanthren-3-ol	36.23	0.78	AMR	MOL004989	(2S)-6-(2,4-dihydroxyphenyl)-2-(2-hydroxypropan-2-yl)-4-methoxy-2,3-dihydrofuro[3,2-g]chromen-7-one	60.25	0.63	RRG
MOL000049	3ß-acetoxyatractylone	54.07	0.22	AMR	MOL004990	3′-Hydroxy-4′-O-Methylglabridin	43.71	0.57	RRG
MOL000072	8ß-ethoxy atractylenolide III	35.95	0.21	AMR	MOL004991	7-Acetoxy-2-methylisoflavone	38.92	0.26	RRG
MOL001792	Liquiritigenin	32.76	0.18	RRG	MOL004996	Gadelaidic acid	30.7	0.2	RRG
MOL000211	Mairin	55.38	0.78	RRG, PRA	MOL000500	Vestitol	74.66	0.21	RRG
MOL002311	Glycyrol	90.78	0.67	RRG	MOL005000	Gancaonin G	60.44	0.39	RRG
MOL000239	Jaranol	50.83	0.29	RRG	MOL005001	Gancaonin H	50.1	0.78	RRG
MOL002565	Medicarpin	49.22	0.34	RRG	MOL005003	Licoagrocarpin	58.81	0.58	RRG
MOL000354	Isorhamnetin	49.6	0.31	RRG	MOL005007	Glyasperins M	72.67	0.59	RRG
MOL000359	Sitosterol	36.91	0.75	RRG, CR, RRP, PRA	MOL005008	Glycyrrhiza flavonol A	41.28	0.6	RRG
MOL003656	Lupiwighteone	51.64	0.37	RRG	MOL005012	Licoagroisoflavone	57.28	0.49	RRG
MOL003896	7-Methoxy-2-methyl isoflavone	42.56	0.2	RRG	MOL005016	Odoratin	49.95	0.3	RRG
MOL000392	Formononetin	69.67	0.21	RRG	MOL005017	Phaseol	78.77	0.58	RRG
MOL000417	Calycosin	47.75	0.24	RRG	MOL005018	Xambioona	54.85	0.87	RRG
MOL004328	Naringenin	59.29	0.21	RRG	MOL005020	Dehydroglyasperins C	53.82	0.37	RRG
MOL004805	Shinflavanone	31.79	0.72	RRG	MOL000098	Quercetin	46.43	0.28	RRG
MOL004806	Euchrenone	30.29	0.57	RRG	MOL000360	Ferulic acid	39.56	0.06	ASR, CR
MOL004808	Glyasperin B	65.22	0.44	RRG	MOL011782	Ligustilide	23.5	0.07	ASR, CR
MOL004810	Glyasperin F	75.84	0.54	RRG	MOL002143	Senkyunolide-C	46.8	0.08	ASR, CR
MOL004811	Glyasperin C	45.56	0.4	RRG	MOL002111	3-Butylidenephthalide	42.44	0.07	ASR, CR
MOL004814	Isotrifoliol	31.94	0.42	RRG	MOL001494	Mandenol	42	0.19	CR
MOL004815	Kanzonol B	39.62	0.35	RRG	MOL002135	Myricanone	40.6	0.51	CR
MOL004820	Kanzonol W	50.48	0.52	RRG	MOL002140	Perlolyrine	65.95	0.27	CR
MOL004824	6-prenylated eriodictyol	39.22	0.41	RRG	MOL002157	Wallichilide	42.31	0.71	CR
MOL004827	Semilicoisoflavone B	48.78	0.55	RRG	MOL000433	Folic Acid	68.96	0.71	CR
MOL004828	Glepidotin A	44.72	0.35	RRG	MOL001918	Paeoniflorigenone	87.59	0.37	PRA
MOL004829	Glepidotin B	64.46	0.34	RRG	MOL001919	Palbinone	43.56	0.53	PRA
MOL004833	Phaseolinisoflavan	32.01	0.45	RRG	MOL001924	paeoniflorin	53.87	0.79	PRA
MOL004835	Glypallichalcone	61.6	0.19	RRG	MOL000492	Cianidanol	54.83	0.24	PRA
MOL004838	Glabrocoumarone A	58.44	0.38	RRG	MOL000748	5-(Hydroxymethyl)-2-furaldehyde	45.07	0.02	RRP
MOL004841	Licochalcone B	76.76	0.19	RRG	MOL001436	Leonuride	2.6	0.33	RRP
MOL004848	Licochalcone G	49.25	0.32	RRG	MOL002819	Catalpol	5.07	0.44	RRP
MOL004849	Licoarylcoumarin	59.62	0.43	RRG	MOL000067	Valine	53.33	0.01	ACC
MOL004855	Licoricone	63.58	0.47	RRG	MOL007579	Hydroxyproline	83.55	0.02	ACC
MOL004856	Gancaonin A	51.08	0.4	RRG	MOL000061	Proline	77.57	0.01	ACC
MOL004857	Gancaonin B	48.79	0.45	RRG	MOL000050	Glycine	48.74	0	ACC
MOL004863	Gancaonin L	66.37	0.41	RRG	MOL000071	Histidine	53.18	0.03	ACC
MOL004864	Gancaonin M	30.49	0.41	RRG	MOL000054	Arginine	47.64	0.03	ACC
MOL004866	Gancaonin O	44.15	0.41	RRG	MOL003971	Threonine	73.52	0.01	ACC
MOL004879	Glycyrin	52.61	0.47	RRG	MOL003969	Serine	98.5	0.01	ACC
MOL004882	Licocoumarone	33.21	0.36	RRG	MOL000052	Glutamic Acid	6.66	0.02	ACC
MOL004883	Licoisoflavone	41.61	0.42	RRG	MOL000042	Alanine	87.69	0.01	ACC
MOL004884	Licoisoflavone B	38.93	0.55	RRG	MOL005449	Methionine	70.87	0.01	ACC
MOL004885	Licoisoflavanone	52.47	0.54	RRG	MOL005448	Leucine	72.92	0.01	ACC
MOL004891	Shinpterocarpin	80.3	0.73	RRG	MOL000068	Isoleucine	59.05	0.02	ACC
MOL004898	5-Prenylbutein	46.27	0.31	RRG	MOL000056	Tyrosine	57.55	0.05	ACC
MOL004903	Liquiritin	65.69	0.74	RRG	MOL000041	Phenylalanine	41.62	0.04	ACC
MOL004904	Licopyranocoumarin	80.36	0.65	RRG	MOL000065	Aspartic Acid	79.74	0.02	ACC
MOL004907	Glyzaglabrin	61.07	0.35	RRG	MOL001780	Tryptophane	75.93	0.08	ACC

OB, oral bioavailability; DL, drug-likeness; GRR, Ginseng Radix et Rhizoma; P, Poria; AMR, Atractylodis Macrocephalae Rhizoma; RRG, Glycyrrhizae Radix et Rhizoma; ASR, Angelicae Sinensis Radix; CR, Chuanxiong Rhizoma; PRA, Paeoniae Radix Alba; RRP, Rehmanniae Radix Praeparata; ACC, Asini Corii Colla.

**Figure 2:**
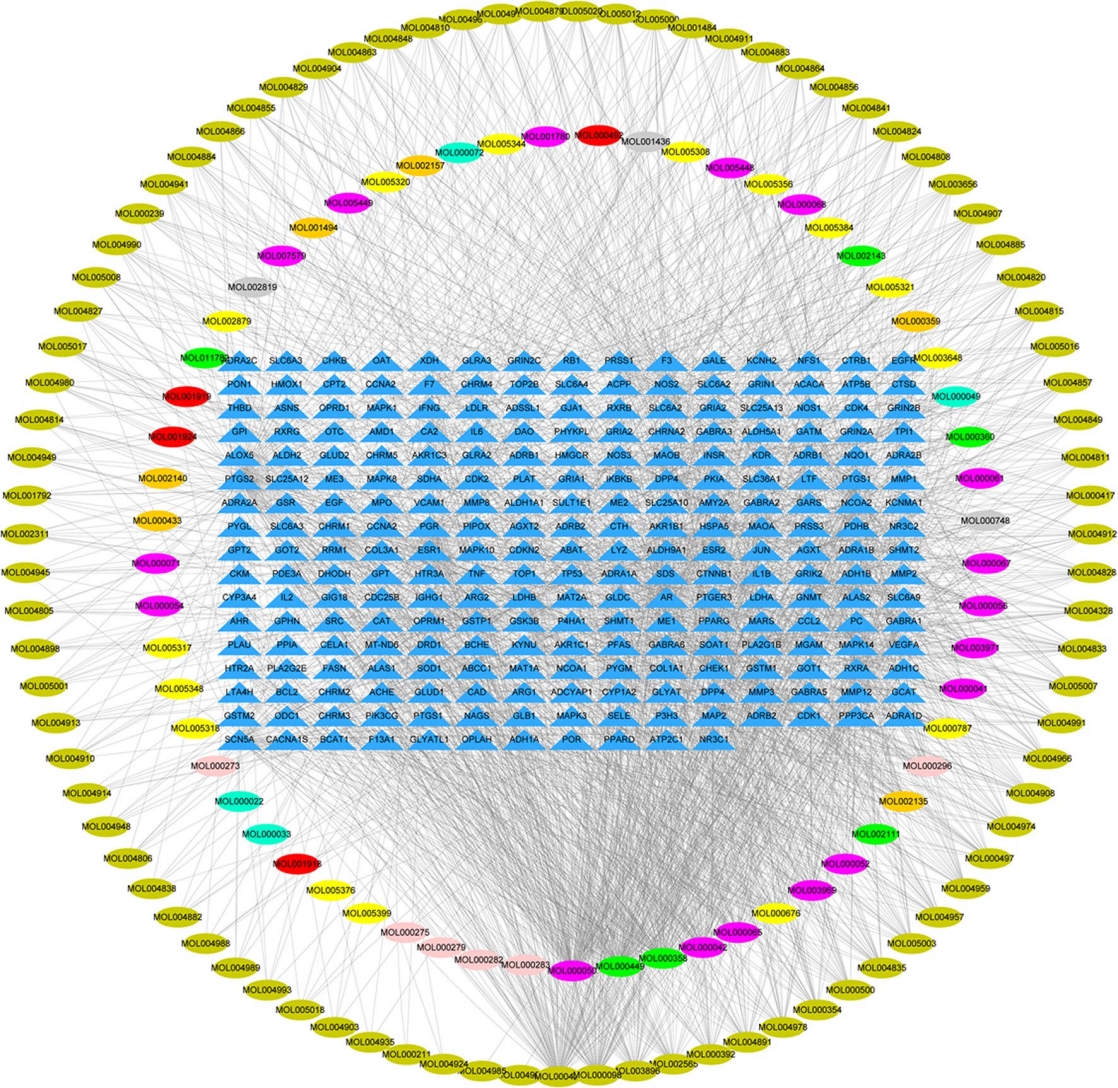
Compound- target network of YQSX. The blue triangles represent targets; the yellow, pink, cyan, kelly green, green, orange, red, gray, and violet ovals represent the compounds from GRR, P, AMR, RRG, ASR, CR, PRA, RRP, and ACC, respectively.

The Compound-target network of YQSX was constructed using the screened compounds and their targets as shown in **Figure 2**. The network contained 399 nods (148 compounds in YQSX and 251 compound targets) and 2,177 edges which indicated the compound-target interactions. One hundred and forty-eight candidate compounds had a median of 12 degrees, which suggested that most compounds of YQSX affected multiple targets. Kaempferol, glycine, and stigmasterol acted on 118, 99 and 87 targets, respectively. And the OB of kaempferol, glycine, and stigmasterol is 41.88, 48.74, and 43.83%, respectively. Therefore, they might be the crucial active compounds of YQSX by reason of their considerable positioning in the network.

### PPI Networks Analysis

PPI operates large-scale biological processes, such as cell-to-cell interactions, metabolic control, and developmental control, and is increasingly regarded as the primary objectives of system biology (Rao et al., 2014). Therefore, PPI networks of YQSX putative targets and ITP-related targets were visualized using PPI data. The PPI network of YQSX putative targets contained 5,959 nodes and 148,332 edges, which represented 5,959 interacting protein and 148,332 interactions. The PPI network of ITP-related targets contained 5,163 nodes and 127,564 edges.

### Identification of Candidate Targets for YQSX Against ITP

**Figure 3:**
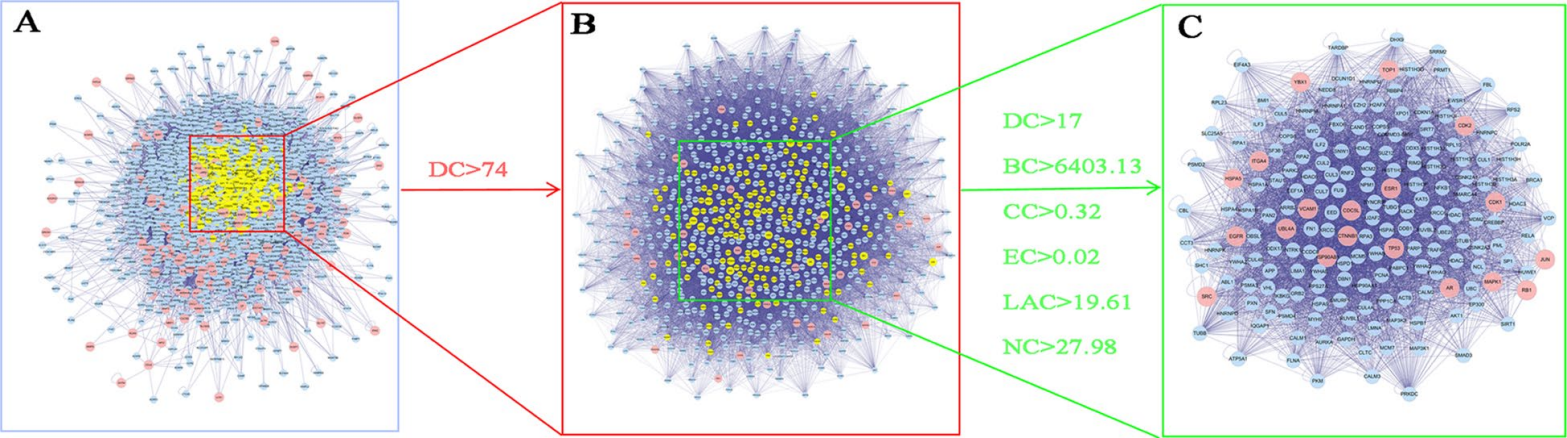
Identification of candidate targets of YQSX against ITP. **(A)** The interactive PPI network of YQSX putative targets and ITP-related targets. **(B)** PPI network of significant proteins extracted from A. **(C)** PPI network of candidate YQSX targets for ITP treatment extracted from B. DC, degree centrality; BC, betweenness centrality; CC, closeness centrality; EC, eigenvector centrality; LAC, local average connectivity-based method; NC, network centrality.

In order to reveal the mechanisms of action underling YQSX’s effects on ITP, the PPI network of YQSX putative targets and the PPI network of ITP-related targets were merged to identify the candidate targets for YQSX against ITP. This network consisting of 3,232 nodes and 95,775 edges was presented in **Figure 3A**. The median degree of all nodes was 37 and the nodes with more than 74 degrees were identified as significant targets according to the previous research (Zhang et al., 2013). A network of significant targets for YQSX against ITP was constructed and it contained 780 nodes and 35,907 edges (**Figure 3B**). The median values of DC, BC, CC, EC, LAC, and NC were 117, 6,403.125, 0.318309, 0.024411, 19.61376, and 27.984, respectively. The candidate targets were further screened and 183 targets with DC > 117, BC > 6,403.125, CC > 0.318309, EC > 0.024411, LAC > 19.61376, and NC > 27.984 were identified (**Figure 3C**). One hundred and eighty-three target genes were eventually identified for YQSX against ITP.

### GO and Pathway Enrichment Analysis

**Figure 4:**
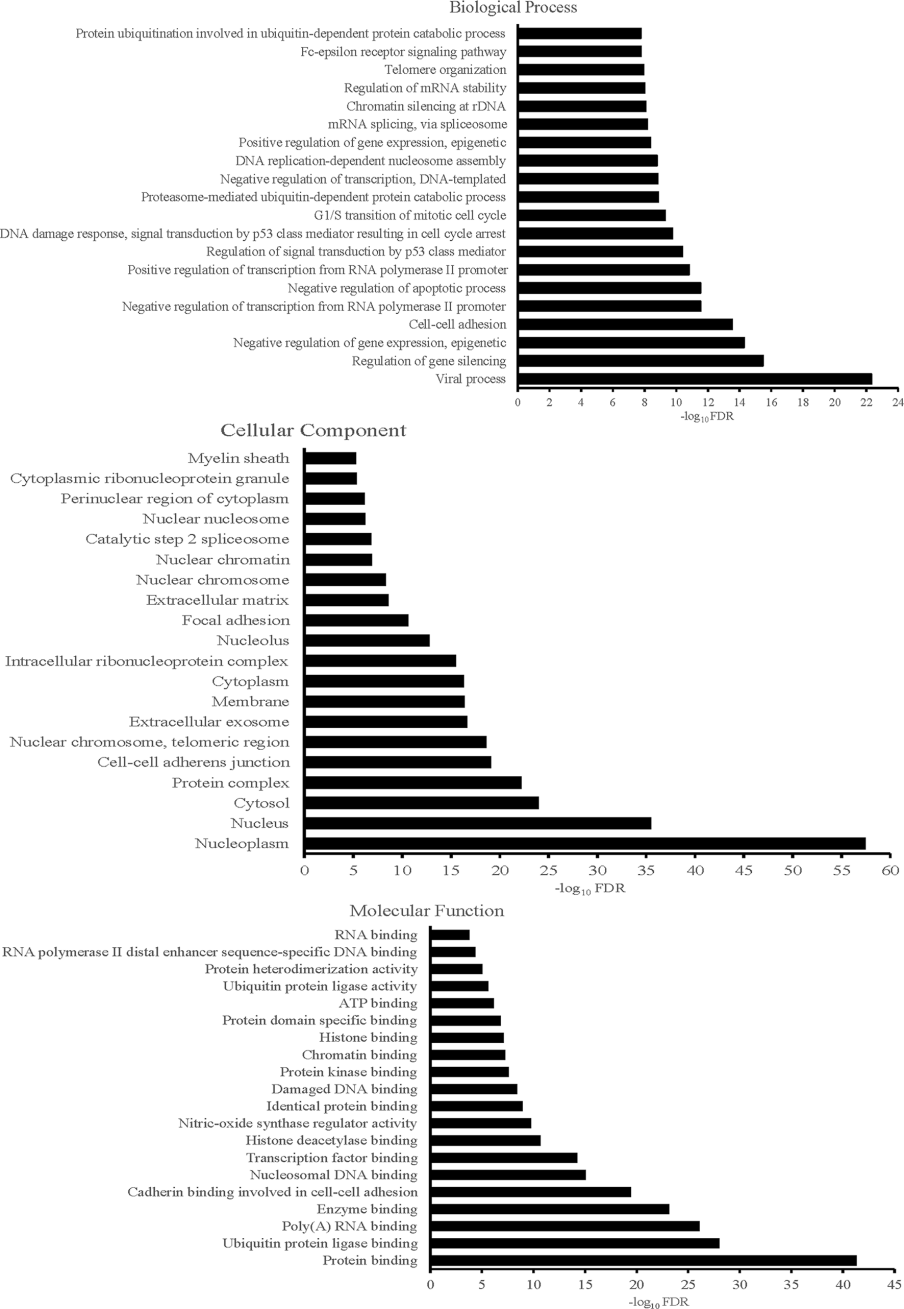
Gene ontology terms of candidate targets of YQSX against ITP. The top 20 GO functional categories with FDR < 0.05 were selected.

DAVID was used to perform GO and KEGG pathway analysis of the 183 candidate targets identified. GO of candidate targets was analyzed based on biological process, cellular component, and molecular function. One hundred seventy-two GO terms were significantly enriched (FDR < 0.05), 98 in biological process, 29 in cellular component, and 45 in molecular function. The data of GO analysis were shown in **Supplementary Table 1**. Top 20 terms were shown in **Figure 4**. The highly enriched GO terms in biological process, cellular component, and molecular function included regulation of gene silencing, regulation of gene expression, nucleoplasm, nucleus, protein binding, and ubiquitin protein ligase binding.

**Figure 5:**
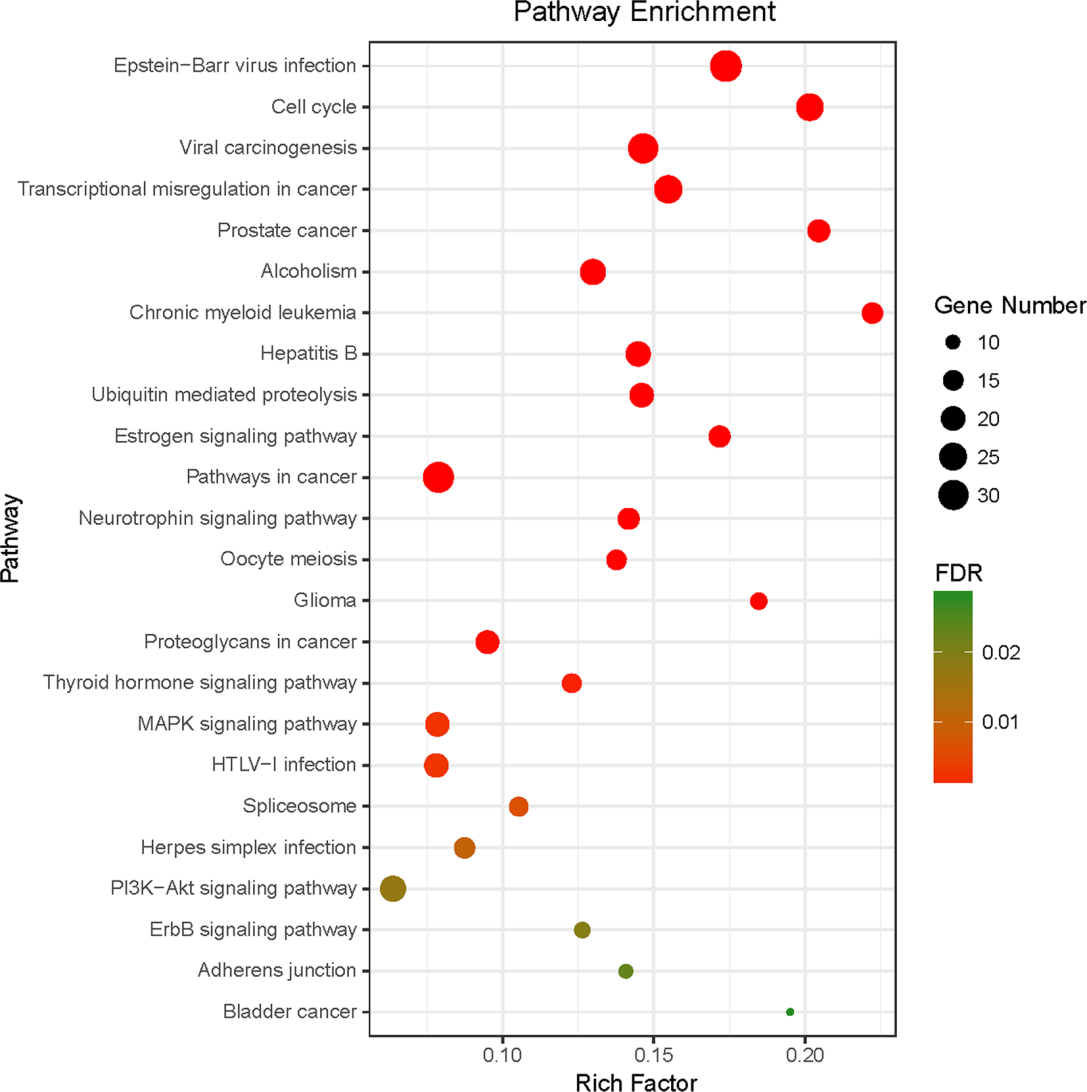
KEGG pathway enrichment of candidate targets of YQSX against ITP. Pathways that had significant changes of FDR <0.05 were identified. Size of the spot represents number of genes and color represents FDR value.

The pathways that were significantly influenced by YQSX in the process of treating ITP were identified by KEGG pathway analysis. Twenty-four significantly enriched pathways (FDR < 0.05) including Epstein-Barr virus infection, cell cycle, estrogen signaling pathway, pathway in cancer, and MAPK signaling pathway were identified. The data of KEGG pathway analysis were shown in **Supplementary Table 2**. As shown in **Figure 5**, size of the spot represented number of genes and color represented FDR value.

### Gene-Pathway Network Analysis

**Figure 6:**
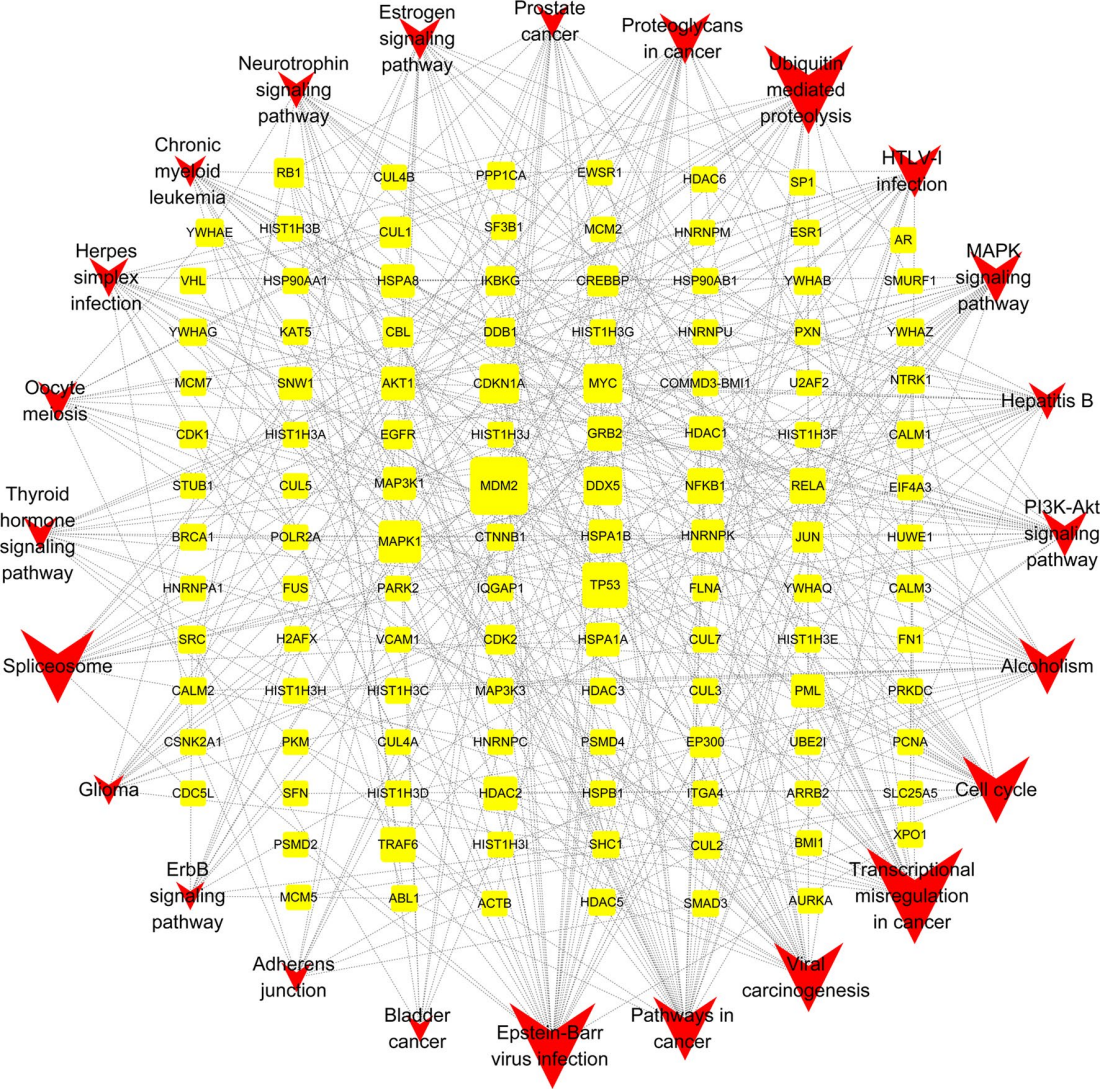
Gene-Pathway Network of YQSX against ITP. The topological analysis of 24 pathways and 115 genes was carried out with betweenness centrality. The yellow squares represent target genes and the red V-shapes represent pathways. Big size represents the larger betweenness centrality.

The gene-pathway network was constructed based on the significantly enriched pathways and genes that regulated these pathways, which was presented in **Figure 6**. The topological analysis of 24 pathways and 115 genes was carried out with BC. The squares represented target genes and the V-shapes represented pathways in the network. The network diagram suggested that MDM2 had the most maximum BC and was the core target gene. Other several genes also had larger BC, such as TP53, MAPK1, CDKN1A, MYC, and DDX5. They might be the key target gens for YQSX against ITP.

## Discussion

The unique medical theory of TCM has been formed and developed over thousands of years in China for the treatment and preventions of diseases. Multiple compatible herbs are usually used as complex herbal formulations to improve therapeutic effect through synergism (Li et al., 2016). In TCM, ITP is thought to be a disease caused by the failure of *spleen* to manage blood. Guipi decoction and BZD were the most common prescribed formulas in the treatment of ITP in TCM. YQSX, the improved formula of BZD, is an empirical formula to treat ITP in TCM clinical practice, and has demonstrated significant clinical effects. Compared with BZD, ACC is added to YQSX. ACC has been shown to enrich the *blood* and to improve hemorheology, hemostasis, and immunological regulation, and its addition has further strengthened the BZD’s effects for the treatment of ITP. TCM adopts a holistic approach focusing on overall functional recovery and elimination of the cause of the disease. The concept of network pharmacology is comparable to TCM theory and is therefore appropriate to be used for the research on unknown components and mechanism of action of complex TCM herbal formulations supported by a variety of databases and software available.

In the present study, a compound-target network of YQSX was constructed using the 148 compounds and 251 compound targets. The results suggested that most compounds of YQSX affected multiple targets, for example, kaempferol, glycine, and stigmasterol acted on 118, 99, and 87 targets, respectively. Therefore, they were very likely to be the crucial pleiotropically active compounds for YQSX. Although the number of putative targets in each single herb was different, the overlapping targets in different herbs were numerous. In another word, multiple compounds of YQSX may have the same target providing synergistic effects. Kaempferol is a representative flavonoid and has been shown to exert multiple pharmacological activities, such as antioxidant, anti-inflammatory, anti-cancer, anti-diabetic, anti-osteoarthritic, and immunomodulatory properties (Tsai et al., 2018; Wang et al., 2018). Lin et al. (2011) reported that kaempferol might be a potent immunosuppressant to decrease the harmful immune responses, including chronic inflammation and autoimmunity. Glycine is an important amino acid contributing to metabolism, growth, development, immunity, cytoprotection, and survival owning to its anti-inflammatory and immunomodulatory properties (Lu et al., 2017; Heidari et al., 2018). Stigmasterol also showed anti-cancer, anti-inflammatory and anti-allergic properties as well as the modulatory effects on immune responses (Antwi et al., 2017). TCM is a highly complex system and contains a large number of constituents. Researchers try to verify even more effective chemical components from TCM through various approaches including network pharmacology. But there has not been a way to recognize the total effective constituents of TCM up till the present moment. It is well known that the effects of TCM on treating diseases are the result of the combination effects of many constituents. In the present study, kaempferol, glycine, and stigmasterol regulated the most targets associated with ITP and all of them have immunomodulatory properties. Although kaempferol, glycine, and stigmasterol are ubiquitous and widely known compounds, there is some evidence for their immunomodulatory effects. In addition, they have high oral bioavailability and kaempferol and stigmasterol came from 3 herbs of YQSX. Therefore, they might be identified as the representative compounds for YQSX.

The PPI networks of YQSX putative targets and ITP-related targets were structured and merged to obtain the candidate targets for YQSX against ITP. In order to get the more accurate targets, 6 parameters including DC, BC, and CC were set to screen nodes and structure a new network. One hundred and eighty-three targets were finally identified and used to carry out the bioinformatic analysis to elucidate the mechanisms underlying the anti-ITP effects of YQSX.

The targets of YQSX against ITP were enriched in biological processes, cellular components, and molecular function by GO enrichment analysis. Results suggested that YQSX regulated some biological processes, such as gene silencing, gene expression, apoptotic process, and signal transduction by p53 class mediator. ITP is an autoimmune disease characterized by an abnormality in T cell immunity and T cell mechanism has been proved to be an important pathophysiologic mechanism in ITP (Jernås et al., 2013; Ji et al., 2014). It has been shown that allogenic T cell responses could be inhibited through the production of immunoregulatory dendritic cells resulted by silencing RelB (Zhang et al., 2010). The expression of CD72 and IL-2 was decreased whilst the IFN-γ/IL-4 expression was increased in ITP patients (Zhou et al., 2012). Apoptotic process plays an important role in maintenance of normal immune system development, and a failure of apoptotic function has been shown to be associated with the pathogenesis of ITP (Qian et al., 2018). It has been found that mesenchymal stem cells from ITP patients showed increased apoptosis and a defect in immunoregulation and the apoptotic rate was decreased by inhibiting the expression of p53 (Zhang et al., 2016). Therefore, YQSX may help to regulate immunological function through intervening these biological/pathological processes. It has been suggested that the pathogenesis of ITP was associated with gene expression, regulation of apoptosis, regulation of cell proliferation, nucleoplasm, transcription factor binding, histone deacetylase binding, protein kinase binding, and core promoter binding (Deng et al., 2017; Zuo et al., 2017), all of which were significantly enriched in the present study. Therefore, YQSX may exert regulatory function in the pathogenesis of ITP and may also affect some cellular components and molecular function including nucleoplasm, nucleus, cytosol, protein binding, enzyme binding, and DNA binding in the treatment of ITP. Studies have found that the ultrastructural abnormalities in cytoplasmic vacuolization, mitochondrial swelling, abnormal chromatin condensation, and increased staining for activated caspase-3 in megakaryocytes also occur in ITP patients (Kistanguri and McCrae, 2013).

TCM is multi-component, multi-target, and multi-pathway. YQSX, as a TCM formula, also has the same characteristic. Therefore, it can be sure that YQSX treats ITP through multi-pathway. In the present study, a total of 24 KEGG pathways including MAPK signaling pathway and PI3K-AKT signaling pathway were significantly enriched. MAPKs can regulate gene expression, immune response, cell proliferation, apoptosis, and response to oxidative stress, which is one of the mechanisms of immune regulation (Li et al., 2017). Research suggested that PI3K-AKT signaling pathways played a role in reducing excessive innate immune responses and crosstalk between MAPK, which was one of the mechanisms to balance the innate immune responses (Rohani et al., 2010). Eltrombopag is a thrombopoietin receptor agonist and has been used to treat the thrombocytopenia of ITP. The signaling mechanisms of eltrombopag are involved in AKT and MAPK pathways, which is similar to that of thrombopoietin (Kim et al., 2018). Therefore, YQSX may regulate immunological function through the related pathways in the process of ITP treatment. In this study, several pathways related to viral also were significantly enriched. The viral infection relates to the genesis of ITP. The body’s autoimmune response is activated when infected with virus, such as Human Immunodeficiency Virus, Hepatitis C Virus, Epstein-Barr virus, Cytomegalovirus, Herpes simplex virus, and Varicella zoster virus, and the autoimmune response will perpetuate itself despite viral clearance (Audia et al., 2017). The autoimmune response triggered by viral infection might be regulated by YQSX through specific pathways, such as Epstein-Barr virus infection, viral carcinogenesis, Hepatitis B, HTLV-I infection, and Herpes simplex infection. In a human study, plasma samples from 74 ITP patients and 58 healthy controls were collected and bioinformatic analysis was carried out. The results indicated that the occurrence of ITP was associated with proteoglycans in cancer, prostate cancer, glioma, thyroid hormone signaling pathway, and estrogen signaling pathway (Zuo et al., 2017). In the present study, the aforementioned pathways were also significantly enriched, which suggested that the regulation of pathways associated with the occurrence of ITP might be one of the mechanisms of YQSX for treatment of ITP. In addition, YQSX may function by regulating other pathways, including cell cycle, and neurotrophin and ErbB signaling pathways. Inhibition of cell cycle caused by selective inhibition of lymphocyte proliferation was found to be beneficial for treating refractory ITP (Grace and Neunert, 2016).

Gene-pathway network was constructed to investigate the core and key target genes for YQSX against ITP. Results suggested that MDM2 had the maximum BC and it might be the core target gene. Other top 5 genes (TP53, MAPK1, CDKN1A, MYC, and DDX5) were selected as the key target gens. MDM2 can negatively regulate p53 which is a central cell cycle regulator and has a negatively regulatory effect on autoimmunity (Liu et al., 2017). MDM2 can block the transactivation domain of p53 and affects gene transcription by inhibiting the ability and then block the progression of cell cycle and promote apoptosis (Wang et al., 2012). MDM2 can regulate a functional autologous immune response; therefore, it is linked to the development of autoimmunity (Mayr et al., 2006). Studies on the role of MDM2 in immune regulation illustrated that inhibition of MDM2 promoted T cell proliferation induced by dendritic cells (Gasparini et al., 2012). It is well-known that MAPKs are an essential regulator of immune responses. The TP53 gene provides instructions to make the p53 protein (Vickers, 2018). The expression and stability of p53 can be promoted by inhibiting PKA and p53 phosphorylation inactivates BCL-XL, leading to platelet apoptosis (Zhao et al., 2017). Research found that CDKN1A has a potential pro-apoptotic effect by reason of arresting cells at G1 or G2/M phases (Hui et al., 2014). CDKN1A protein is also a p53 transcriptional target and can activate cell cycle checkpoints, promote DNA repair, downregulate apoptosis, and trigger a senescence-like growth arrested response, all of which play an important role in the network of DNA damage surveillance (Mirzayans and Murray, 2016). MYC has been suggested to directly coordinate the immune response through regulating the immune checkpoints expression (Casey et al., 2017). It has been widely accepted that the role of Th17 cells in ITP is a possible pathogenic factor and a potential therapeutic target of ITP (Ye et al., 2015). DDX5 was found to control the differentiation of Th17 cells at steady state and autoimmunity (Huang et al., 2015).

The mechanisms of action and molecular targets of YQSX for ITP were explored using a network pharmacology approach in this study. Kaempferol, glycine, and stigmasterol regulated the most targets associated with ITP. YQSX may regulate immunological function through the specific biological processes including gene silencing, gene expression, apoptotic process, and signal transduction by p53 class mediator and the related pathways including MAPK signaling pathway and PI3K-AKT signaling pathway. In addition, YQSX may exert its regulatory function in the pathogenesis of ITP and the regulation of pathways including proteoglycans in cancer, prostate cancer, glioma, and thyroid hormone and estrogen signaling pathways which are associated with the occurrence of ITP. MDM2, TP53, MAPK1, CDKN1A, MYC, and DDX5 were the key target gens in the gene network of YQSX for treatment of ITP. The network pharmacology appears to be a suitable approach for the study of complex TCM formulations.

## Author Contributions

YJ and NL performed main analysis and drafted the manuscript. JL and XH designed the research. SZ helped for introduction and discussion. DC assisted in the preparation of the manuscript. All authors wrote, read, and approved the manuscript.

## Funding

This work was supported by the National Basic Research Program of China (973 Program, 2015CB554403 and 2015CB554405) and National Natural Science Foundation of China (81803772).

## Conflict of Interest

NL was employed by Beijing Increase Research for Drug Efficacy and Safety Co., Ltd.

The remaining authors declare that the research was conducted in the absence of any commercial or financial relationships that could be construed as a potential conflict of interest.

The handling editor declared a shared affiliation, though no other collaboration, with one of the authors YJ at the time of the review.

## Supplementary Material

Table S1 The data of GO enrichment analysis including biological process, cellular component, and molecular function.

Table S2 The data of KEGG pathway enrichment analysis.
